# E-cadherin and NF-κB expression in the vagina after ovarian ischemia and reperfusion

**DOI:** 10.1590/acb391724

**Published:** 2024-04-15

**Authors:** Arzu Yurci, Fuat Zaman, Umut Sarı, Engin Deveci

**Affiliations:** 1 İstanbul Bahcelievler Memorial Hospital – Department of Obstetrics and Gynecology – İstanbul, Turkey.; 2 Diyarlife Hospital – Department of Obstetrics and Gynecology – Diyarbakır, Turkey.; 3 Dicle University – Faculty of Medicine – Department of Histology and Embryology – Diyarbakır, Turkey.

**Keywords:** Vagina, Ovary, Ischemia, Reperfusion, Rats

## Abstract

**Purpose:**

To investigate inflammation and cell adhesion molecules in the vagina after ovarian ischemia-reperfusion (IR) injury.

**Methods:**

20 Wistar albino female rats were divided into two groups: control, and IR groups. In IR group, blood flow was restricted for 2 hours for ovarian ischemia. Then, tissues were re-blood 2 hours for reperfusion. Vagina tissues were excised and processed for histopathological analysis. Histopathological and biochemical follow-ups were performed.

**Results:**

Both malondialdehyde and myeloperoxidase values were increased in IR group compared to control group. Glutathione content was decreased in IR group compared to control group. Epithelial degeneration, inflammation, dilatation, and nuclear factor-κB (NF-κB) expression were increased in IR group compared to control group. E-cadherin expression was significantly decreased in IR group. In the IR group, E-cadherin showed a positive reaction in adenomas, gland-like cryptic structures, cellular junctions with clustered inflammatory cells. In the IR group, NF-κB expression was increased in basement membrane, inflammatory cells, in blood vessels.

**Conclusions:**

Ovarian ischemia caused degeneration of epithelial cells in the vaginal region and disruptions in the cell junction complex, which leads to activation of E-cadherin and NF-κB signaling pathway and alterations in reproductive and embryonal development in the vaginal region.

## Introduction

Ovarian ischemia is a gynecological emergency that occurs with torsion due to interruption of blood flow. Ovarian torsion is based on the principle that the arterial and venous vessels of the ovary make partial or full rotation on their axis, preventing blood flow. Nausea, vomiting and acute abdominal pain with positive peritoneal findings are among the symptoms of ovarian torsion. As a result of adnexal torsion, arterial blood flow is interrupted firstly, and venous blood flow is interrupted secondly due to edema formation. Ischemia is the direct result of adnexal torsion. In untreated conditions, ischemia may result in necrosis of the ovary, fallopian tube, or the entire adnexal structure^1^^,^ 2.

Adnexal cysts have been shown to be risk factors for ovarian torsion in developing conditions such as ovarian hyperstimulation or pregnancy, such as ovarian enlargement and hypoxia of the ovarian propria and infundibulopelvic ligament one. During the ischemia process, increased neutrophil infiltration and excessive production of reactive oxygen species (ROS) cause increased damage in the reperfusion stage. Increased malondialdehyde (MDA) occurs during lipid peroxidation. Cell membrane lipids are structures affected by ROS and are important criteria to indicate the level of oxidative damage^3^. Glutathione, the most important intracellular antioxidant that protects cells against oxidative damage, has been reported to decrease significantly in the case of ischemia. In the inflammation that occurs after ischemia in a tissue, reperfusion becomes the tissue sensitive to this damage.

**Figure 1:**
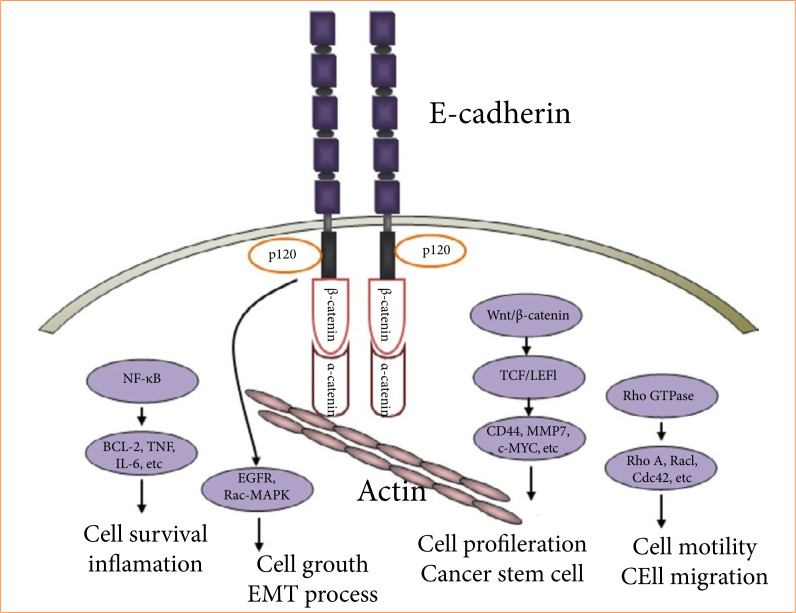
E-cadherin regulated signaling pathways. Activation of these pathways leads to an increase in cell proliferation, decrease in cell apoptosis, cell migration, and inflammation associated cancer development.

Epithelial cadherin (E-cadherin) is an important protein involved in cell-to-cell junctional complexes of epithelial cells, responsible for adhesion, morphogenesis, and tissue organization. It has been suggested that any alteration in E-cadherin expression may be associated with tumor invasion and metastasis in gastric and ovarian cancers and some other tumors (Fig. 1). Estradiol is known to be a potent stimulator of E-cadherin expression in the ovary and uterus^4^^,^ 5.

E-cadherin: epithelial cadherin; NF-κB: nuclear factor kappa B; BCL-2: B-cell lymphoma-2; TNF: tumor necrosis factor-alpha; IL: interleukin; EGFR: epidermal growth factor receptor; MAPK: mitogen-activated protein kinases; EMT: epithelial-to-mesenchymal transition; TCF: T cell factor; LEF1: lymphoid enhancer factor family 1; CD44: cluster of differentiation 44; MMP7: matrix metalloproteinase-7; c-MYC: cellular-myelocytomatosis ; Rho GTPases: Ras homolog guanosine 5’-triphosphates. Source: Elaborated by the authors.

Nuclear factor-κB (Nf-κB) is a ubiquitous inducible transcription factor involved in inflammatory and stress responses within the immune system. Inflammation is a process that causes tissue damage with increased oxidative stress, and interleukin (IL)-1β and IL-6 are the main proinflammatory cytokines that indicate the presence of inflammation^6^^,^ 7.

Our study aimed to investigate the effect of inflammation in the vagina, which occurs as a result of oxidative stress after ischemia and reperfusion, at the immunohistochemical level.

## Methods

### Experimental design

#### Animals

All experiments performed in this study were approved by the Ethics Committee for Animal Experimentation, Dicle University. Twenty Wistar albino female rats (weighing 220–250 g) were bought, housed in separate cages at 23 ± 2°C, 12 hours light/12 hours dark period at 45–55% humidity and fed with standard pellet and water. Estrous cycles of rats were determined by vaginal smear taken at 6–12-hour intervals. After cell examination under microscope, 20 female rats in estrous cycle were included in the experiment. Before starting the experimental procedure, 90-mg / kg intramuscular ketamine hydrochloride (Ketalar; Pfizer, Istanbul, Turkey) and 8-mg / kg xylazine (Rompun; Bayer, Istanbul, Turkey) were given for general anesthesia. Rats were divided into two groups (10 rats per group), and the following procedures were applied to the groups.

### Experimental groups

- Control group (n = 10): no treatment was applied to animals. Only the abdomen was opened with a surgical protocol, and the abdominal folds were closed without any further intervention;
- Ischemia-reperfusion (IR) group (n = 10): the abdominal area was opened with a 2-cm midline incision. Ischemia was created for 2 hours with a disposable Bulldog clamp on ovarian tissues. Then, the ovaries were placed back to their normal positions, in their anatomical location, and the blood flow was reperfused for 2 hours.

### Histological tissue processing and immunohistochemistry protocol

Vagina tissues were fixed in zinc-formal for 24 hours. Five-micron sections were cut from the paraffin blocks with a rotary microtome and stained with hematoxylin-eosin dye to examine the tissue histopathology. Sections were deparaffinized in xylene and brought to distilled water. Endogenous peroxidase activity was blocked in 0.1% hydrogen peroxide for 20 min. Ultra V block was applied for 15 min. Tissue sections were incubated with NF-κB (#sc-8008, Santa Cruz Biotechnology, United States of America) and E-cadherin (#sc- sc-8426, Santa Cruz Biotechnology, United States of America) antibody and overnight. The sections were washed three times for 5 min in phosphate buffered saline (PBS) and then incubated with biotinylated secondary antibody for 25 min.

After washing with PBS, streptavidin peroxidase was applied to the sections for 15 min. The sections were washed three times for 5 min in PBS. Diaminobenzidine was applied to the sections for up to 15 min as a chromogen. The control slides were prepared using the same procedure, without primary antibodies. Counterstaining was done using Harris’s hematoxylin for 45 seconds, dehydrated through ascending alcohol and cleared in xylene. The slides were mounted with Entellan (#107961, Merck, Germany) and examined under a light microscope (Olympus, Germany).

## Results

### Biochemical findings

Both malondialdehyde (MDA) and myeloperoxidase (MPO) values were increased in IR group compared to control group, and the increase was statistically significant. Glutathione (GSH) content was decreased in IR group compared to control group, and the decrease was statistically significant. Epithelial degeneration, inflammation, dilatation, and NF-κB expression were increased in IR group compared to control group, and the increase was statistically significant. E-cadherin expression was significantly decreased in IR group. Biochemical and histopathological scores are shown in Table 1.

**Table 1:** Biochemical (MDA, GSH and MPO), histopathological (epithelial degeneration, inflammation, dilatation) and immunohistochemical scores (E-cadherin and NF-κB expression) of control and IR.

Parameter	Groups	Median (min–max)	Mann-Whitney U test
MDA	(1) Control	5.78 (4.45–7.45)	p = 0.011
	(2) IR	9.34 (8.88–14.65)	
MPO	(1) Control	2.45 (1.24–5.83)	p = 0.023
	(2) IR	7.03 (5.92–10.45)	
GSH	(1) Control	1.46 (1.35–1.69)	p = 0.003
	(2) IR	0.42 (0.25–0.68)	
Dilatation	(1) Control	0.5 (0.00–1.00)	p = 0.001
	(2) IR	3.0 (2.00–3.00)	
Inflammation	(1) Control	0.5 (0.00–1.00)	p = 0.001
	(2) IR	3.00 (2.00–3.00)	
Epithelial degeneration	(1) Control	0.5 (0.00–1.00)	p = 0.001
	(2) IR	3.00 (2.00–3.00)	
E-cadherin expression	(1) Control	2.5 (2.00–3.00)	p = 0.001
	(2) IR	0.00 (0.00–1.00)	
NF-κB expression	(1) Control	0.5 (0.00–3.00)	p = 0.001
	(2) IR	3.00 (2.00–3.00)	

MDA: malondialdehyde: MPO: myeloperoxidase; GSH: glutathione; E-cadherin: epithelial cadherin; NF-κB: nuclear factor kappa B; IR: ischemia-reperfusion. Source: Elaborated by the authors.

### Histopathological findings

**Figure 2:**
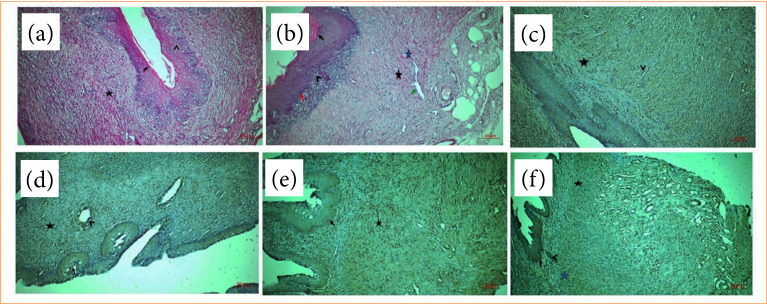
Hematoxylin and eosin staining **(a)** control group: towards the lumen, the epithelium is slightly keratinized (black arrow), the cells of the stratified epithelium extending towards the cervix are polygonal (arrowhead), connective tissue cell infiltrates (star) in the lamina propria; **(b)** IR group: small adenomas (black arrow) surrounded by fibrinoid elements, hyperplasic cells in the basement membrane (red arrow), inflammatory cell infiltration in the papillary region (arrowhead), deterioration in collagenous structures (black star), dilatations in blood vessels (green arrow), bleeding foci (blue star); E-cadherin immune staining; **(c)** control group: positive E-cadherin reaction in basement membrane (black arrow), collagen fibrils (arrowhead), negative E-cadherin expression in lamina propria (star); **(d)** IR group: E-cadherin positivity in the inner parts of adenomas (black arrow), in gland-like cryptic structures (arrowhead) at the bottom, and in areas in which inflammatory cells (star) are present, NF-κB immune staining; **(e)** control group: positive in cells located in the basement membrane (black arrow), NF-κB positive in connective tissue cells (star); **(f)** IR group: NF-κB positive reaction of basement membrane some cell membranes (black arrow), aggregate inflammatory cell communities (arrowhead), inflammatory cells (black star), connective tissue cells (blue star). Scale bar: 50 μm, magnification: 20X.

IR: ischemia-reperfusion; E-cadherin: epithelial cadherin; NF-κB: nuclear factor kappa B. Source: Elaborated by the authors.

In the section of control group, it was observed that the epithelium was slightly keratinized towards the lumen. The cells of the stratified epithelium extending towards the cervix were observed in oval shape and polygonal appearance. Small blood vessels with nerve endings were seen in the papilla area. Widespread connective tissue cell infiltrations and irregularly arranged collagen fibers were observed in the lamina propria. It was observed that the muscle bundles were located in a circular manner (Fig. 2a).

In the section of IR group, small adenomas surrounded by keratinized and fibrinoid elements and some cells with atypical appearance were detected, especially in the epithelium. Hyperplasic cells were seen in the basement membrane in the lower part. A dense inflammatory cell infiltration and small adenomas extending downwards were seen in the papillary region. Deteriorations were detected in the collagenized structures towards the lower sides. Dilatations, freely dispersed erythrocyte infiltrates and bleeding foci were observed in the blood vessels in between. Solitary lymphocytic cells were observed around the blood vessels. It was observed that inflammation increased, apoptosis accelerated, and epithelial cells became indistinct (Fig. 2b).

In the control group, it was observed that there was positivity in places along the basement membrane, but there was a negative E-cadherin reaction in general and there was no deterioration in the cells. On the lower side, integrity was preserved in the lamina propria, and E-cadherin expression continued to be negative. E-cadherin reaction was positive in collagen fibrils, fibroblasts, and muscle bundles (Fig. 2c).

In the IR group, it was determined that the cells in the inner parts of the adenomas were atypical, and small apoptotic cell infiltrates were increased towards the upper sides of the adenomas and E-cadherin showed a positive reaction. It was determined that there was a very significant thinning in the epithelium. A positive E-cadherin reaction was also detected in gland-like cryptic structures at the bottom. E-cadherin positivity was also detected in the areas in which the apoptotic process started, especially in the villous structure of the deteriorated interconnection complexes and in the areas with clustered inflammatory cells (Fig. 2d).

In the examination of control vagina, NF-κB expression was observed in some cells with an important structure in the basement membrane that showed mitogenic activity. Solitary dispersed cell infiltrates are inflammation due to increased secretion originating from the superficial epithelial area. The vessels were normal, not overly dilated. Moderate NF-κB in connective tissue cells infiltration was seen (Fig. 2e).

In the IR group, NF-κB expression was positive in some cell membranes with the basement membrane border, in small aggregates due to the intense cytokine activity. Inflammatory cell populations showed NF-κB positivity. Blood vessels showed NF-κB positive reaction in external inflammatory cells with endothelial cell dysfunction. Many cells with positive reactions were found in connective tissue cells. Increased inflammation was observed (Figure 2f).

## Discussion

Ischemia causes the insufficient delivery of oxygen and other metabolites by the circulation to the tissues, leading to cell death and organ failure. Reperfusion of the ischemic tissue sometimes may give the more harm than ischemia itself. During IR injury, production of ROS increases. MDA is an indicator of lipid peroxidation in tissues. High level of MDA is related to oxidative damage. The cell scavenges these harmful molecules by its antioxidant enzymes such as superoxide dismutase (SOD), GSH and catalase (CAT)^8^^,^ 9.

Celik et al. found that MDA level was increased in both serum plasma and ovarian tissues in an experimental ovarian IR injury^10^. Toprak et al. found that MDA was increased and GSH was decreased in ovarian IR animal model^11^. In our study, both MDA and MPO values were increased in IR group compared to control group, and the increase was statistically significant. GSH content was decreased in IR group compared to control group, and the decrease was statistically significant. Epithelial degeneration, inflammation, dilatation, and NF-κB expression were increased in IR group compared to control group, and the increase was statistically significant. E-cadherin expression was significantly decreased in IR group (Table 1).

Ovarian ischemia reperfusion causes many pathologies in ovarian tissues, as well as remote organs such fallopian tubes, uterine and cervix. Aktaş et al. studied ovarian IR and found intense fibrosis, vascular dilatation and congestion, stromal inflammation in ovarian tissues after IR^12^. Peker et al. investigated IR injury in ovarian tissues and revealed that IR caused edema, inflammation, congestion, degenerated follicles, and cells with pyknotic nuclei^13^.

Eser et al. graded the ovarian tissues histologically after IR injury and recorded that the histological scores of ovarian tissues with IR was lower than control group^14^. In our study, in the section of control group, blood vessels had the normal characteristics of the epithelial layer, irregularly arranged collagen fibers were observed in the lamina propria with normal muscles (Fig. 2). In the section of IR group, small adenomas surrounded by keratinized and fibrinoid elements, dense inflammatory cell infiltration, degenerated collagen fibers, and hemorrhage were observed. It was observed that inflammation increased, apoptosis accelerated, and epithelial cells became indistinct (Fig. 2, Table 1).

E-cadherin is an important molecule in the maintenance of epithelial integrity. Intracellular junctions are major contributors to the epithelial barrier and are composed of E-cadherin-mediated intercellular attachments. Damage to the epithelium may result in the loss of E-cadherin expression in the cell membrane and intercellular junction^15^^,^ 16. Goto et al. studied lung IR injury to investigate the role of E-cadherin. They found that E-cadherin concentration and expression are increased and undergo cleavage during IR^17^. Menezes et al. studied renal IR. They revealed that E-cadherin is differentially expression in medulla and cortex. E-cadherin expression was increased in after IR injury^18^. In our study, in the control group, E-cadherin expression was positive in basement membrane, but generally negative. In the IR group, E-cadherin showed a positive reaction in adenomas, gland-like cryptic structures, cellular junctions with clustered inflammatory cells (Fig. 2, Table 1).

NF-κB has been shown to play a key role in inflammatory response, neuronal survival and signaling. It is a transcription factor that induces many vital genes^19^. NF-κB seems to be involved in pathogenesis of ovarian IR injury. Ozturk et al. studied role of NF-κB in ovarian IR. The authors found that, after IR, oxidative stress and inflammation were increased and these cellular events upregulated the expression of NF-κB in IR groups^20^. A similar study was done by Deniz et al. In their ovarian IR injury model on animals, they found NF-κB expression was increased in ovarian tissues^21^. In the examination of control vagina, NF-κB expression was observed in basement membrane, cell infiltrates and in the vessels. In the IR group, NF-κB expression was increased in basement membrane, inflammatory cells, in blood vessels (Fig. 2, Table 1).

Melatonin is an indoleamine produced by the pineal gland and known to have a strong antioxidant effect. Its free radical scavenging feature has been used in animal experiments to provide advantages in different organ transplants. In addition, melatonin has been demonstrated in experimental studies to have anti-inflammatory and antioxidant properties that may affect tissue growth and apoptosis.

Shiroma et al. found that melatonin promoted faster resumption of the estrous cycle compared to control and increased the expression of mature follicles, collagen type I, von Willebrand factor, Ki-67, and cleaved caspase-3 on estrogen receptors in the corpus lutea and ovaries. It has also been stated that there was a decrease in apoptosis in follicles, corpus lutea and collagen type III with TUNEL^22^.

A study investigating the relationship between melatonin and organ transplantation found that heart transplantation surgical procedure, immunosuppression, and grafting did not affect melatonin secretion in rodents, but there was a significant decrease in melatonin levels during the kidney transplantation procedure in patients with renal failure. It was also suggested that melatonin application in experimental models reduces organ rejection and increases transplant success^23^.

In a review study on melatonin and its use in ovarian transplantation, melatonin was reported to improve various graft characteristics, such as morphology, apoptosis, immunological reaction, revascularization, oxidative stress, and survival rate. The antioxidative and antiapoptotic properties of melatonin have apparently been stated to have positive effects on ovarian graft activity. It was concluded that, despite positive results, further studies in humans are needed to solidify its use, as ovarian transplantation for fertility preservation has slowly moved from the experimental phase to the clinical setting^24^.

## Conclusion

As a result of ovarian ischemia, degeneration of epithelial cells in the vaginal region and disruptions in the cell junction complex increased secretion. It was thought that the activation of the E-cadherin and NF-κB signaling pathway and the progression of the apoptotic process may cause changes in reproductive development and embryonal development in the vaginal region as a result of ovarian ischemia.

## Data Availability

All data sets were generated or analyzed in the current study.
